# The added value of hypertonic saline solution to furosemide monotherapy in patients with acute decompensated heart failure: A meta‐analysis and trial sequential analysis

**DOI:** 10.1002/clc.24033

**Published:** 2023-06-20

**Authors:** Carlos Diaz‐Arocutipa, Jack Denegri‐Galvan, Lourdes Vicent, Marcos Pariona, Mamas A. Mamas, Adrian V. Hernandez

**Affiliations:** ^1^ Vicerrectorado de Investigación Universidad San Ignacio de Loyola Lima Peru; ^2^ Deparment of Cardiology Hospital Nacional Daniel Alcides Carrión Callao Peru; ^3^ Cardiology Department Hospital Universitario 12 de Octubre and Instituto de Investigación Sanitaria Hospital 12 de Octubre (imas12) Madrid Spain; ^4^ Centro de Investigación Biomédica en Red Enfermedades Cardiovasculares (CIBERCV) Madrid Spain; ^5^ Department of Cardiology Hospital Nacional Edgardo Rebagliati Martins Lima Peru; ^6^ Keele Cardiovascular Research Group, Centre for Prognosis Research Keele University Keele UK; ^7^ Health Outcomes, Policy, and Evidence Synthesis (HOPES) Group University of Connecticut/Hartford Hospital Evidence‐Based Practice Center Hartford CT USA

**Keywords:** acute heart failure, furosemide, hypertonic saline solution, systematic review

## Abstract

We assessed the effects of hypertonic saline solution (HSS) plus furosemide versus furosemide alone in patients with acute decompensated heart failure (ADHF). We searched four electronic databases for randomized controlled trials (RCTs) until June 30, 2022. The quality of evidence (QoE) was assessed using the GRADE approach. All meta‐analyses were performed using a random‐effects model. A trial sequential analysis (TSA) was also conducted for intermediate and biomarker outcomes. Ten RCTs involving 3013 patients were included. HSS plus furosemide significantly reduced the length of hospital stay (mean difference [MD]: −3.60 days; 95% confidence interval [CI]: −4.56 to −2.64; QoE: moderate), weight (MD: −2.34 kg; 95% CI: −3.15 to −1.53; QoE: moderate), serum creatinine (MD: −0.41 mg/dL; 95% CI: −0.49 to −0.33; QoE: low), and type‐B natriuretic peptide (MD: −124.26 pg/mL; 95% CI: −207.97 to −40.54; QoE: low) compared to furosemide alone. HSS plus furosemide significantly increased urine output (MD: 528.57 mL/24 h; 95% CI: 431.90 to 625.23; QoE: moderate), serum Na^+^ (MD: 6.80 mmol/L; 95% CI: 4.92 to 8.69; QoE: low), and urine Na^+^ (MD: 54.85 mmol/24 h; 95% CI: 46.31 to 63.38; QoE: moderate) compared to furosemide alone. TSA confirmed the benefit of HSS plus furosemide. Due to the heterogeneity in mortality and heart failure readmission, meta‐analysis was not performed. Our study shows that HSS plus furosemide, compared to furosemide alone, improved surrogated outcomes in ADHF patients with low or intermediate QoE. Adequately powered RCTs are still needed to assess the benefit on heart failure readmission and mortality.

## Graphical abstract

Hypertonic saline solution (HSS) plus furosemide compared to furosemide alone improves outcomes in acute decompensated heart failure (ADHF) patients. CV, cardiovascular; HF, heart failure; MD, mean difference; RCTs, randomized controlled trials; RR, risk ratio.

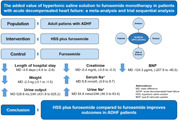

## 1 INTRODUCTION

Currently, acute decompensated heart failure (ADHF) represents a high burden of morbidity and mortality worldwide.^1^ Treatment of fluid overload is a major issue in the management of ADHF patients, with loop diuretics (e.g., furosemide) being the cornerstone therapy.^2^, ^3^ In some patients, usual doses of loop diuretics are not enough to relieve symptoms of congestion, and up to half of the patients leave the hospital with persistent fluid overload which is associated with rehospitalizations and higher mortality.^4^ This insufficient response, known as diuretic resistance, has been shown to contribute to worsening heart failure during hospitalization, prolonged lengths of stay, and increased mortality.^5^, ^6^, ^7^ Adding other diuretics, such as thiazides or metolazone, acting in different sites in the nephron is a common practice trying to address diuretic resistance.^1^ However, this strategy requires careful monitoring of electrolytes, especially sodium and potassium, and renal function. Thus, use of hypertonic saline solution (HSS) in combination with furosemide has emerged as a novel therapeutic approach.^8^

The addition of HSS to furosemide is based on its property to recall free water contained in the interstitial spaces, increasing the intravascular compartment during diuretic therapy.^9^ Subsequently, HSS can prevent the decline in effective arterial circulating volume and the consequent possible decrease in renal blood flow, being the main reason to be considered in scenarios like refractory decompensated heart failure.^8^ Therefore, we performed a systematic review and meta‐analysis to assess the effects of HSS plus furosemide versus furosemide alone in ADHF patients.

## 2 METHODS

This systematic review was reported following the 2020 Preferred Reporting Items for Systematic Reviews and Meta‐Analyses (PRISMA) statement.^10^ Ethical approval was not required because this study will retrieve and synthesize data from already published studies.

### 2.1 Search strategy

We searched in the following electronic databases from inception to January 29, 2021, with an update on June 30, 2022: PubMed, Embase, Scopus, and Web of Science. The complete search strategy is available in Supporting Information: Table S1. There were no restrictions on language or publication date. We also performed a hand search of reference list of all included studies and relevant review articles to identify other potentially eligible studies.

### 2.2 Eligibility criteria

The inclusion criteria were as follows: (i) randomized controlled trials (RCTs) involving adult patients (≥18 years old) with ADHF, (ii) RCTs evaluating any dose and duration of HSS plus furosemide as the intervention group, (iii) any dose and duration of furosemide as the control group, and (iv) RCTs that report at least one evaluated outcome at any length of follow‐up. Observational studies, case reports, case series, systematic reviews, preprints, conference abstracts, and editorials were excluded.

### 2.3 Selection of studies

We downloaded all articles from electronic search to EndNote X8 and duplicate records were removed. All unique articles were uploaded to Rayyan (https://rayyan.qcri.org/) for the study selection process. Titles and abstracts were independently screened by two review authors (Carlos Diaz‐Arocutipa and Jack Denegri‐Galvan) to identify relevant studies. Furthermore, the same review authors (Carlos Diaz‐Arocutipa and Jack Denegri‐Galvan) independently examined the full‐text of selected studies and registered reasons for the exclusion. Any disagreement on title/abstract and full‐text selection was resolved by consensus.

### 2.4 Outcomes

The outcomes were classified as follows: clinical outcomes (all‐cause mortality, cardiovascular mortality, and heart failure readmission), intermediate outcomes (length of hospital stay, urine output, and weight), and biomarker outcomes (serum Na^+^, urine Na^+^, serum creatinine, and type‐B natriuretic peptide [BNP]). We used the study‐reported definitions for all outcomes.

### 2.5 Data extraction

The information from each selected study was independently extracted by two review authors (Carlos Diaz‐Arocutipa and Jack Denegri‐Galvan) using a standardized data extraction form in an Excel spreadsheet that was previously piloted. Any disagreement was resolved by consensus. If additional data was needed, we contacted the corresponding author through email. The following data were extracted: first author name, publication year, country, study design, sample size, population, age, sex, comorbidities, left ventricular ejection fraction (LVEF), intervention group, comparator group, and clinical, intermediate, and biomarker outcomes.

### 2.6 Risk of bias assessment

Two review authors (Carlos Diaz‐Arocutipa and Jack Denegri‐Galvan) independently assessed the risk of bias in each study using the Cochrane risk of bias (RoB) tool 2.0.^11^ Any disagreement was resolved by a third author (Adrian V. Hernandez). The RoB 2.0 tool evaluates five domains: randomization process, deviations from intended interventions, missing outcome data, measurement of the outcome, and selection of the reported result. Overall, each RCT was judged as having a low, some concerns, or a high risk of bias.

### 2.7 Assessment of the quality of evidence

We used the grading of recommendations, assessment, development and evaluation (GRADE) approach to evaluate the quality of evidence for each outcome.^12^ The GRADE methodology examines the following five categories: risk of bias, consistency, indirectness, imprecision, and reporting bias. Each RCT started as high‐quality evidence and will be downgraded based on the criteria described above. The quality of evidence was categorized as high, moderate, low, or very low. We generated the summary of findings (SoF) table using the GRADEpro software.

### 2.8 Statistical analyses

All meta‐analyses were conducted using the inverse‐variance random‐effects model. Treatment effects were expressed as relative risk (RR) with their 95% confidence interval (CI) for dichotomous outcomes and mean difference (MD) with their 95% CI for continuous outcomes.^13^ Only final values for each group were compared for urine output, serum Na^+^, urine Na^+^, serum creatinine, and BNP. For weight, the difference between final and baseline values was compared. Only studies that evaluated outcomes with similar follow‐up times and cointerventions were pooled. Heterogeneity was evaluated using the chi‐squared test (threshold *p* < .10) and the *I* ^2^ statistic, with values of *I* ^2^ > 60% corresponding to substantial statistical heterogeneity. No meta‐analysis was performed for clinical outcomes due to the high variability in cointerventions (dietary sodium content and daily dose of furosemide) administered postdischarge and time of follow‐ups across studies. Thus, only a narrative synthesis of these results was conducted. Publication bias was assessed only if >10 RCTs were available per outcome. Subgroup analyses by the type of population (refractory vs. unselected patients with heart failure), duration of HSS plus furosemide (<6 vs. ≥6 days), a daily dose of furosemide (>200 mg/day vs. ≤200 mg/day), and country (Italy vs. non‐Italian countries) were conducted. The duration of HSS plus furosemide and daily dose of furosemide were categorized by their median values. We also performed a sensitivity analysis including only RCTs with a low risk of bias. In addition, we analyzed results using cumulative meta‐analysis according to the publication year. The *meta* package from R 4.2.0 software (R Foundation for Statistical Computing) was used for all meta‐analyses. A two‐tailed *p* < .05 was considered statistically significant.

Furthermore, we conducted a trial sequential analysis (TSA) to evaluate the random errors due to multiple testing and sparse data, and to calculate the required information size.^14^ Our calculation, defined a *priori*, was based on the autogenerated empirical data according to the data input for continuous outcomes, two‐sided type I error of 5%, and statistical power of 80%. We also calculated the TSA‐adjusted CI for all outcomes. This analysis was performed using the TSA software version beta 0.9.5.10 (Copenhagen Trial Unit).

## 3 RESULTS

### 3.1 Study selection

**Figure 1:**
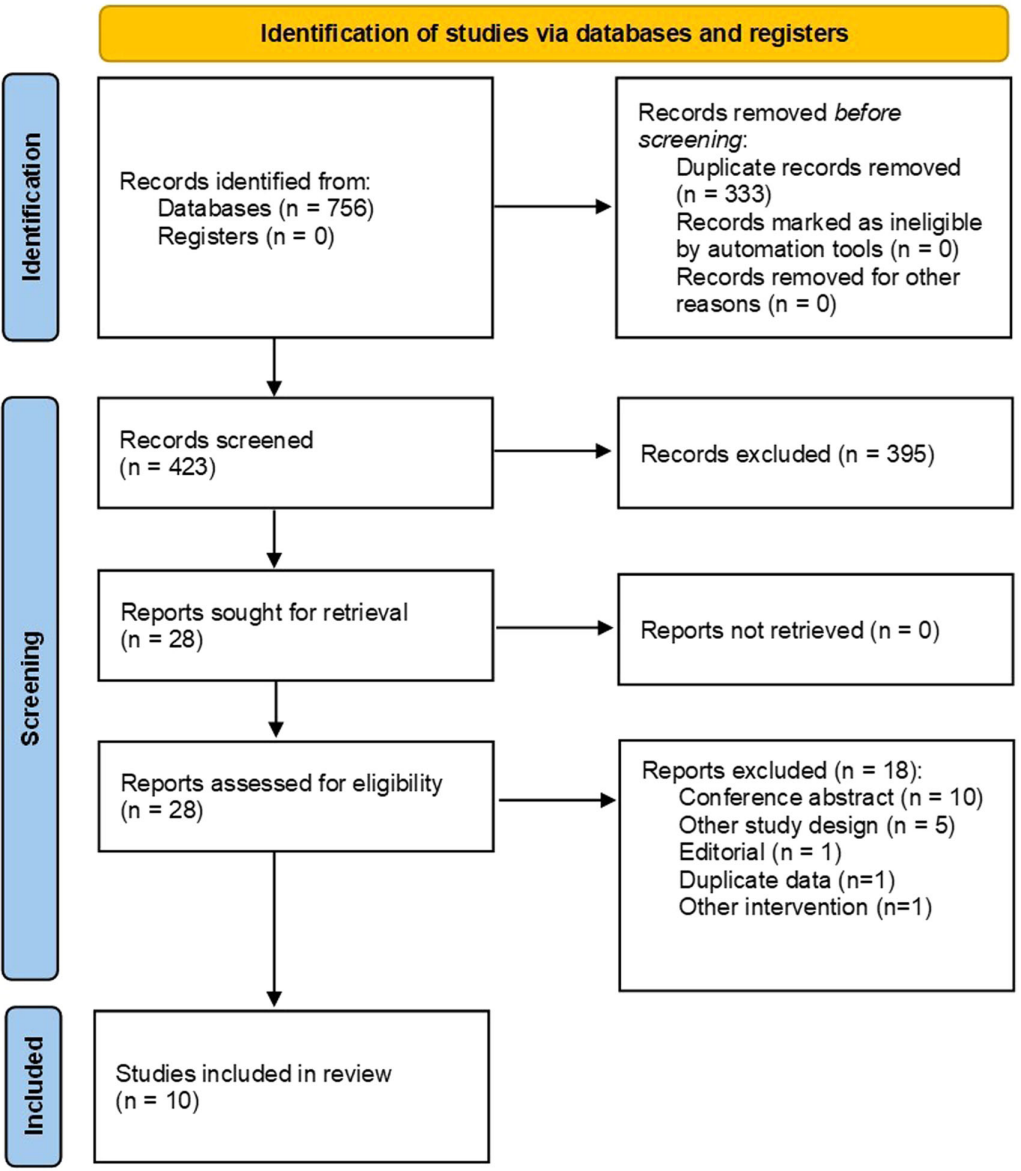
Flow diagram of study selection.

Our electronic search yielded 756 articles. After the removal of 333 duplicates, 423 articles underwent title/abstract screening, of which 28 articles were selected for the full‐text screening. Eighteen articles were excluded by the following reasons: conference abstract (*n* = 10), other study design (*n* = 5), editorial (*n* = 1), duplicate data (*n* = 1), and other intervention (*n* = 1). Finally, 10 articles were included (Figure 1).^15^, ^16^, ^17^, ^18^, ^19^, ^20^, ^21^, ^22^, ^23^, ^24^

### 3.2 Trial characteristics

The main characteristics of the 10 RCTs (*n* = 3013) are summarized in Table 1. The mean age was 70 years and 60% were men across trials. A total of six out of 10 studies were conducted in Italy and the rest in Brazil, Japan, Turkey, and China. Follow‐up time ranged from 24 h to 55 months. The range of sample sizes per study varied between 28 and 1927 patients. The study conducted by Paterna et al. provided most of the population included in the analysis (1927 of 3013 subjects, 64%).^21^ In six studies, the patients were blinded to the treatment. While in five studies, it was the treating physicians. Only in one study^15^ were the investigators blinded and three studies were open label. In half of the studies, only hospitalized patients with refractory ADHF were included, while in the rest, unselected cases were included. In seven studies, the duration of the HSS treatment was <6 days. Mean LVEF ranged from 24% to 56%, with mean LVEF < 40% in eight studies. Comorbidities were reported in eight studies, although information was mostly limited. The most common etiology of heart failure was ischemic cardiomyopathy with a prevalence ranging from 42% to 62%. Mean baseline serum Na^+^ ranged from 135 to 139 mmol/L. The daily dose of administered furosemide in both groups varied between 500 to 2000 mg/day, which corresponded to the trials conducted in Italy, while in non‐Italian trials, varied between 40 and 200 mg/day. Information on the timing of outcome assessment for each study is available in Supporting Information: Table S2.

**Table 1:** Main characteristics of included studies.

Study	Country	Population	Follow‐up duration	Intervention group	Control group	Arms	Sample size	Mean age^a^	Male (%)
Licata et al.^16^	Italy	Patients with refractory uncompensated heart failure with NYHA class IV and LVEF < 35%	7‐55 months (31 ± 14 months)	IV 30 min infusion of furosemide (500–1000 mg) plus HSS (150 mL of 1.4%–4.6% NaCl) bid for 6–12 days	IV 30 min infusion of furosemide (500–1000 mg) bid for 6–12 days	HSS + furosemide	53	74.7 ± 8	62
						Furosemide	54	74.5 ± 6	65
Paterna et al.^20^	Italy	Patients with refractory uncompensated heart failure with NYHA class IV and LVEF < 35%	30 days after discharge	IV 30 min infusion of furosemide (500–1000 mg) plus HSS (150 mL of 1.4%–4.6% NaCl) bid for 4–6 days	IV bolus of furosemide (500‐1000 mg) bid for 4–6 days	HSS + furosemide	48	74.7 ± 8	63
						Furosemide	46	74.5 ± 6	65
Parrinello et al.^19^	Italy	Patients with refractory uncompensated heart failure with NYHA class IV and LVEF < 40%	6 days	IV 20 min infusion of furosemide (250 mg) plus HSS (150 mL of 3.0% NaCl) bid for 6 days	IV bolus of furosemide (250 mg) bid for 6 days	HSS + furosemide	66	75.6 ± 7	64
						Furosemide	67	76.3 ± 9	66
Paterna et al.^21^	Italy	Patients with refractory uncompensated heart failure with NYHA class IV and LVEF < 40%	31–83 months (57 ± 15 months)	IV 30 min infusion of furosemide (250 mg) plus HSS (150 mL of 1.4%–4.6% NaCl) bid until clinical compensation	IV bolus of furosemide (250 mg) bid until clinical compensation	HSS + furosemide	953	74.7 ± 11	63
						Furosemide	974	73.4 ± 13	63
Parrinello et al.^18^	Italy	Patients with acute decompensated heart failure with NYHA class III/IV and LVEF < 45%	Until discharge	IV 30 min infusion of furosemide (250 mg) plus HSS (150 mL of 1.4%–4.6% NaCl) bid until clinical compensation	IV bolus of furosemide (250 mg) bid until clinical compensation	HSS + furosemide	122	74.9 ± 10.9	59
						Furosemide	126	72 ± 8.4	60
Issa et al.^15^	Brazil	Patients with uncompensated heart failure with LVEF < 40%	69.5 (32.2–167.7) days	Furosemide bolus plus IV 60 min infusion of HSS (100 mL of 7.5% NaCl) bid for 3 days	Furosemide bolus plus IV 60 min infusion of 100 mL of NaCl 0.9% bid for 3 days	HSS + furosemide	20	53.3 ± 13	95
						Furosemide	12	41.5 ± 13.1	58
Okuhara et al.^17^	Japan	Patients with acute descompensated heart failure and NYHA class III/IV	24 h	IV furosemide (40 mg/day) plus continous IV infusion of HSS (500 mL of 1.7% NaCl) qd for 24 h	Continuous IV infusion of furosemide (40 mg/day) plus 500 mL 5% glucose qd for 24 h	HSS + furosemide	22	71 ± 11	73
						Furosemide	22	73 ± 10	64
Yayla et al.^24^	Turkey	Patients with acute decompensated heart failure patients with reduced or preserved LVEF	2 days	IV 30 min infusion of furosemide (160 mg) plus HSS (1.4%–7.5% NaCl) for 2 days	IV bolus of furosemide (80 mg) bid for 2 days	HSS + furosemide	14	70.6 ± 8.2	64
						Furosemide	14	71.7 ± 10.7	50
Wan et al.^23^	China	Patients with refractory descompensated heart failure with NYHA class III and LVEF < 40%	4 years	IV 60 min infusion of furosemide (100 mg) plus HSS (100 mL of 2.8% NaCl) bid until clinical compensation	IV furosemide (100 mg) bid until clinical compensation	HSS + furosemide	132	60.6 ± 10.1	40
						Furosemide	132	61.2 ± 10	36
Tuttolomondo et al.^22^	Italy	Patients with acute decompensated heart failure and LVEF < 40%	6 days	IV 30 min infusion of furosemide (120–250 mg) plus HSS (150 mL of 1.4%–4.6% NaCl) bid for 6 days	IV 30 min infusion of furosemide (120–250 mg) bid for 6 days	HSS + furosemide	68	77.9 ± 9.3	57
						Furosemide	68	74.5 ± 6	41

Abbreviations: HSS, hypertonic saline solution, IV, intravenous; LVEF, left ventricular ejection fraction; NYHA, New York Heart Association.

^a^ Data are presented as mean ± standard difference or median (interquartile range).

### 3.3 Risk of bias assessment

Overall, five RCTs were judged as some concerns as the risk of bias (Supporting Information: Figure S1). Five RCTs showed some concerns in the deviations from the intended interventions, two RCTs showed concerns in the measurement of the outcome, and two RCTs showed some concerns in the selection of the reported result. The other five RCTs were judged as low risk of bias.

### 3.4 Clinical outcomes

Only four RCTs evaluated the effect of HSS plus furosemide compared to furosemide alone on all‐cause mortality, cardiovascular mortality, and readmissions for heart failure.^16^, ^20^, ^21^, ^23^ The timing of clinical outcomes assessment ranged from 30 days to 57 months across studies (Supporting Information: Table S2). Licata et al. reported a significantly lower mortality rate (45.3% vs. 87%, *p* < .001) and heart failure readmissions (47.2% vs. 79.6%, *p* < .05) in patients receiving HSS plus furosemide.^16^ Similarly, Paterna et al. found lower mortality (12.9% vs. 23.8%, *p* < .0001) and heart failure readmissions (18.5% vs. 34.2%, *p* < .0001) in the HSS plus furosemide group.^20^, ^21^ Finally, Wan et al. also showed a reduction in mortality (16.5% vs. 31.9%, *p* < .01) and mean time to readmission for heart failure (31.84 vs. 15.6 months, *p* < .01) in the HSS plus furosemide group.^23^

### 3.5 Intermediate outcomes

#### 3.5.1 Length of hospital stay

**Figure 2:**
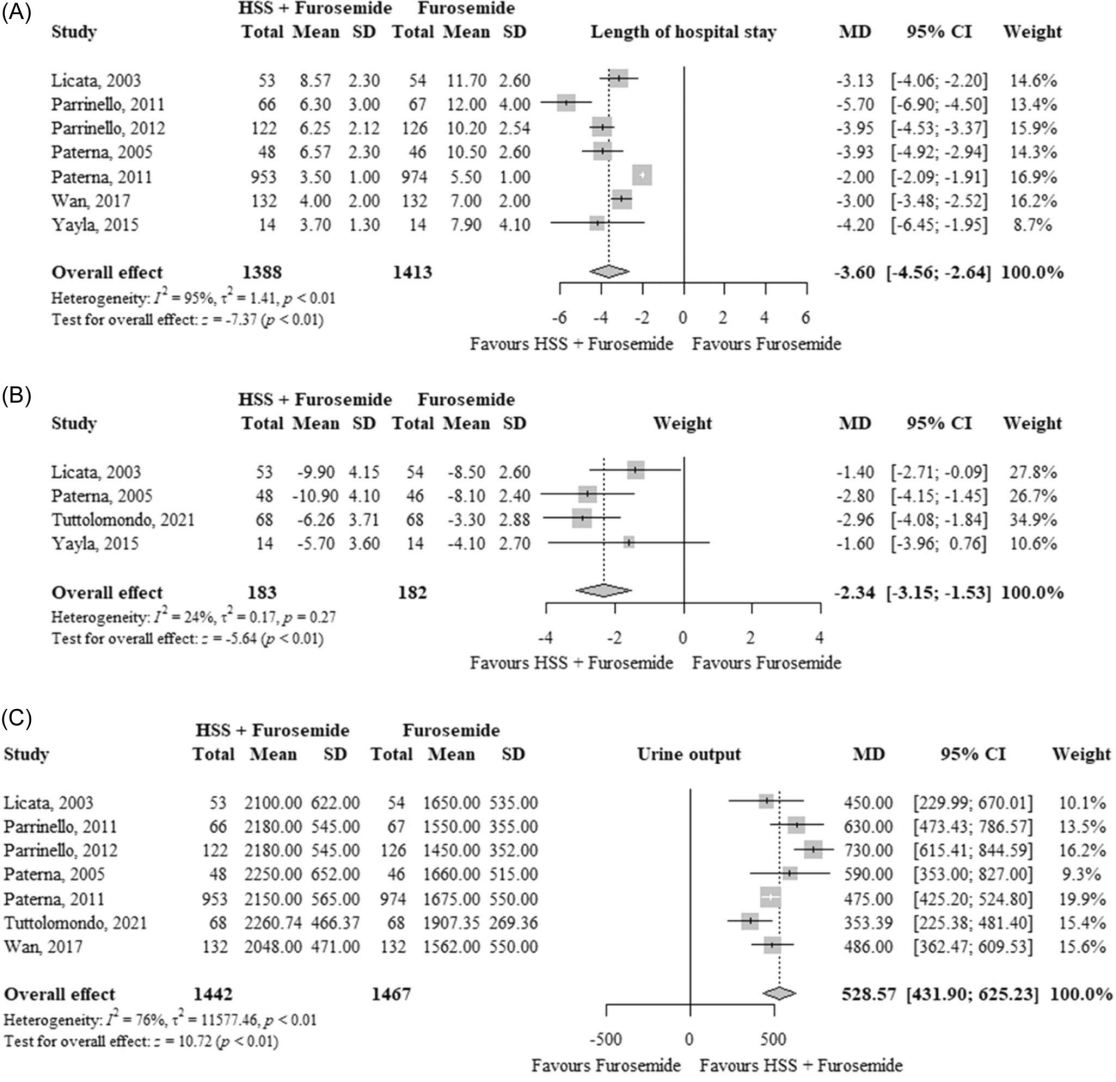
Effects of HSS plus furosemide versus furosemide on (A) length of hospital stay in days, (B) weight in kg, and (C) urine output (mL/24 h). CI, confidence interval; HSS, hypertonic saline solution; MD, mean difference; SD, standard deviation.

In seven RCTs (*n* = 2801), HSS plus furosemide significantly reduced the length of hospital stay (MD: −3.60 days; 95% CI: −4.56 to −2.64; TSA‐adjusted CI: −4.83 to −2.37; *I* ^2^ = 95%) compared to furosemide alone (Figure 2). The quality of evidence was moderate (Table 2).

**Table 2:** Grading of recommendations, assessment, development, and evaluation (GRADE) summary of findings.

Outcomes	Anticipated absolute effects (95% CI)^a^		Relative effect (95% CI)	Number of participants (studies)	Quality of evidence (GRADE)
	Risk with Furosemide	Risk with HSS + Furosemide			
Length of hospital stay follow‐up: range 4–12 days	The mean length of hospital stay was **9.25** days	MD **3.6 days lower** (4.56 lower to 2.64 lower)	‐	2801 (7 RCTs)	⊕⊕⊕◯Moderate^b^
Weight follow‐up: range 2–12 days	The mean weight was −**6** kg	MD **2.34 kg lower** (3.15 lower to 1.53 lower)	‐	365 (4 RCTs)	⊕⊕⊕◯Moderate^c^
Urine output follow‐up: range 4–12 days	The mean urine output was **1636.33** mL/24 h	MD **528.57 mL/24 h higher** (431.9 higher to 625.23 higher)	‐	2909 (7 RCTs)	⊕⊕⊕◯Moderate^d^
Serum creatinine follow‐up: range 1–12 days	The mean serum creatinine was **1.59** mg/dL	MD **0.41 mg/dL lower** (0.49 lower to 0.33 lower)	‐	2613 (8 RCTs)	⊕⊕◯◯Low^b^, ^e^
Serum Na+follow‐up: range 1–12 days	The mean serum Na+ was **133.50** mmol/L	MD **6.8 mmol/L higher** (4.92 higher to 8.69 higher)	‐	2877 (9 RCTs)	⊕⊕◯◯Low^d^, ^e^
Urine Na+ follow‐up: range 1–12 days	The mean urine Na+ was **101.25** mmol/24 h	MD **54.85 mmol/24 h higher** (46.31 higher to 63.38 higher)	‐	2261 (4 RCTs)	⊕⊕⊕◯Moderate^c^, ^e^
BNP follow‐up: range 4–10 days	The mean BNP was **637.80** pg/mL	MD **124.26 pg/mL lower** (207.97 lower to 40.54 lower)	‐	2565 (5 RsCTs)	⊕⊕◯◯Low^c^, ^e^

*Note*: GRADE working group grades of evidence. **High certainty**: we are very confident that the true effect lies close to that of the estimate of the effect. **Moderate certainty**: we are moderately confident in the effect estimate: the true effect is likely to be close to the estimate of the effect, but there is a possibility that it is substantially different. **Low certainty**: our confidence in the effect estimate is limited: the true effect may be substantially different from the estimate of the effect. **Very low certainty**: we have very little confidence in the effect estimate: the true effect is likely to be substantially different from the estimate of effect. Abbreviations: BNP, type‐B natriuretic peptide; CI, confidence interval; MD, mean difference; RCTs, randomized controlled trials; RR, risk ratio.

^a^ **The risk in the intervention group** (and its 95% confidence interval) is based on the assumed risk in the comparison group and the relative effect of the intervention (and its 95% CI).

^b^ Three studies had some concerns as risk of bias.

^c^ Two studies had some concerns as risk of bias.

^d^ Four studies had some concerns as risk of bias.

^e^ *I* ^2^ > 60%.

#### 3.5.2 Weight

In four RCTs (*n* = 365), HSS plus furosemide significantly reduced weight (MD: −2.34 kg; 95% CI: −3.15 to −1.53; TSA‐adjusted CI: −3.25 to −1.39; *I* ^2^ = 24%) compared to furosemide alone (Figure 2). The quality of evidence was moderate (Table 2).

#### 3.5.3 Urine output

In seven RCTs (*n* = 2909), HSS plus furosemide significantly increased urine output (MD: 528.57 mL/24 h; 95% CI: 431.90–625.23; TSA‐adjusted CI: 430.25–626.88; *I* ^2^ = 76%) compared to furosemide alone (Figure 2). The quality of evidence was moderate (Table 2).

### 3.6 Biomarker outcomes

#### 3.6.1 Serum creatinine

**Figure 3:**
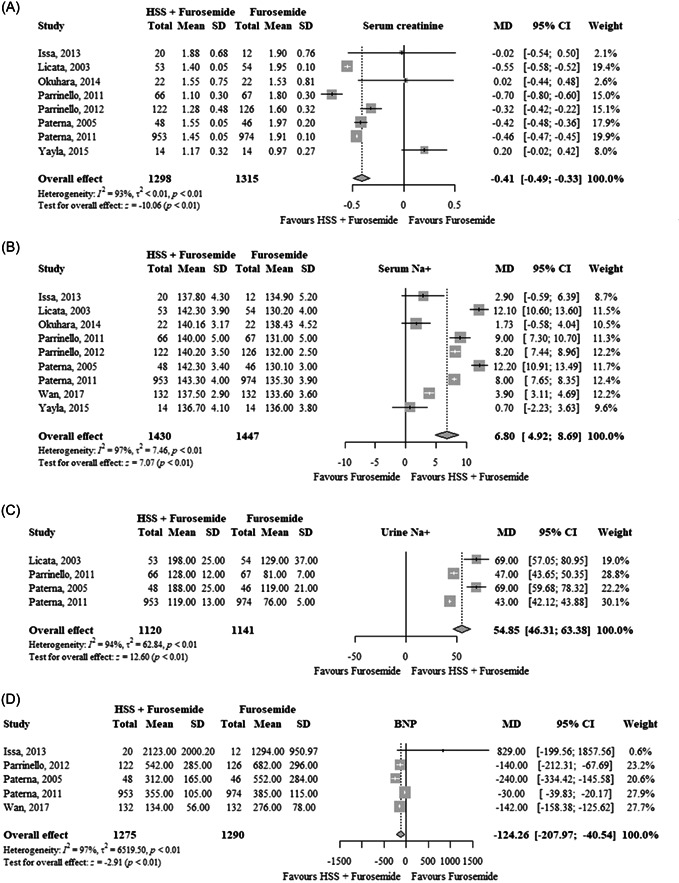
Effects of HSS plus furosemide versus furosemide on (A) serum creatinine in mg/dL, (B) serum Na^+^ in mmol/L, (C) urine Na^+^ in mmol/24 h, (D) BNP in pg/mL, and (E) systolic blood pressure in mmHg. BNP, B‐type natriuretic peptide; CI, confidence interval; HSS, hypertonic saline solution; MD, mean difference; SD, standard deviation.

In eight RCTs (*n* = 2613), HSS plus furosemide significantly reduced serum creatinine (MD: −0.41 mg/dL; 95% CI: −0.49 to −0.33; TSA‐adjusted CI: −0.51 to −0.31; *I* ^2^ = 93%) compared to furosemide alone (Figure 3). The quality of evidence was low (Table 2).

#### 3.6.2 Serum Na^+^

In nine RCTs (*n* = 2877), HSS plus furosemide significantly increased serum Na^+^ (MD: 6.80 mmol/L; 95% CI: 4.92−8.69; TSA‐adjusted CI: 4.48−9.13; *I* ^2^ = 97%) compared to furosemide alone (Figure 3). The quality of evidence was low (Table 2).

#### 3.6.3 Urine Na^+^

In four RCTs (*n* = 2261), HSS plus furosemide significantly increased urine Na^+^ (MD: 54.85 mmol/24 h; 95% CI: 46.31−63.38; TSA‐adjusted CI: 46.23−63.46; *I* ^2^ = 94%) compared to furosemide alone (Figure 3). The quality of evidence was moderate (Table 2).

#### 3.6.4 BNP

In five RCTs (*n* = 2565), HSS plus furosemide significantly reduced BNP (MD: −124.26 pg/mL; 95% CI: −207.97 to −40.54; TSA‐adjusted CI: −79.93 to −44.20; *I* ^2^ = 97%) compared to furosemide alone (Figure 3). The quality of evidence was low (Table 2).

### 3.7 Trial sequential analysis

For all outcomes, the trial sequential monitoring boundaries were crossed by the cumulative *z*‐curves and the TSA‐adjusted CIs showed a significant beneficial effect of HSS plus furosemide over furosemide alone (Supporting Information: Figures S2 and S3). The diversity‐adjusted relative information size was only reached for the following outcomes: length of hospital stay, weight, urine output, serum Na^+^, and urine Na^+^.

### 3.8 Cumulative meta‐analysis

The cumulative meta‐analysis showed no significant change over time for the effects of HSS plus furosemide versus furosemide alone on most outcomes (serum creatinine, serum Na^+^, and urine Na^+^) (Supporting Information: Figures S4 and S5). For BNP, there was an oscillation near the null across the initial years. After adding the last published trial in 2017, the effect was significant.

### 3.9 Subgroup analyses

Subgroup analysis according to the type of population (refractory vs. unselected patients) showed that the interaction test was significant only for serum creatinine (*p* < .01) and serum Na^+^ (*p* = .04) (Supporting Information: Table S3). Serum creatinine was significantly reduced only in refractory ADHF patients and serum Na^+^ was significantly increased only in the same subgroup.

Subgroup analysis according to the duration of HSS plus furosemide (<6 vs. ≥6 days) showed that the interaction test was significant only for serum creatinine (*p* < .01) serum Na^+^ (*p* < .01), although the effect remained significant in all strata (Supporting Information: Table S4).

Subgroup analysis according to the daily dose of furosemide (>200 mg/day vs. ≤200 mg/day) or country (Italy vs. non‐Italian countries) showed that the interaction test was significant only for serum creatinine (*p* < .01) serum Na^+^ (*p* < .01). Serum creatinine was significantly reduced only in patients treated with a daily dose of furosemide >200 mg/day and in studies performed in Italy. Serum Na^+^ was significantly increased in all strata (Supporting Information: Tables S5 and S6).

### 3.10 Sensitivity analysis

Sensitivity analysis including only trials with a low risk of bias showed that the results were consistent with the main analysis for all outcomes (Supporting Information: Table S7).

## 4 DISCUSSION

This meta‐analysis, including 10 RCTs and ~3000 patients with HF, shows that treatment with intravenous HSS plus furosemide was associated with favorable responses across several surrogate efficacy endpoints, with no signals of safety based on low to moderate quality evidence (see Graphical abstract).

The administration of HSS is not used routinely in the management of ADHF patients. Current heart failure clinical practice guidelines dedicate too little attention to this therapy.^25^ However, there is evidence that supports its efficacy and safety in ADHF patients.^15^, ^16^, ^17^, ^18^, ^19^, ^20^, ^21^, ^22^, ^23^, ^24^ The mechanisms underlying the effectiveness of HSS in decongesting patients with acute HF are diverse, including a rapid increase in plasma sodium and osmolality, with a rise in intravascular volume and renal perfusion, but are mainly focused on renal physiology.^26^ A neurohormonal effect inhibiting the deleterious action of the renin‐angiotensin system has been suggested.^27^ This hypothesis has been supported by plasma determination of values of BNP and also other inflammatory and fibrotic parameters (suppression of tumorigenicity 2, inflammatory cytokines [IL‐6]), which were lower in patients receiving HSS.^20^, ^22^

Diuretic resistance is a condition defined by an inability to increase fluid and sodium excretion despite an increase in loop diuretic dose, which is insufficient to relieve volume overload, peripheral edema, or pulmonary congestion.^28^, ^29^ The physiology of diuretic resistance is also complex and not fully understood.^28^, ^29^, ^30^ It has been suggested that renal function (mainly glomerular filtration) would play a limited role in diuretic resistance, with sodium handling at the renal tubules being the most relevant mechanism.^30^ Therefore, diuretic combination strategies targeting sodium reabsorption at different tubular levels are highly effective in patients with diuretic resistance, but at the cost of worsening kidney function and notable electrolyte abnormalities that harm patients’ outcomes.^31^ HSS administration improved the performance of loop diuretic therapy, as reflected in increased urinary volume and urinary sodium, and achieved a greater patient weight loss.^32^ To that effect, HSS is an adjunctive useful measure in patients with diuretic resistance. Furthermore, a trend towards a lower increase in creatinine levels was also observed in patients who received HSS.^21^

Hyponatremia is a factor that markedly worsens the prognosis in ADHF patients, and its treatment can be complex.^33^, ^34^, ^35^, ^36^ Given the effectiveness of HSS in dealing with this problem,^37^, ^38^ it could be a very useful tool, although it requires careful administration and close monitoring to avoid hypercorrection of plasma sodium levels. A previous trial addressing the efficacy of tolvaptan (an aquaretic that antagonizes vasopressin‐2 receptor action that is approved for the treatment of hyponatremia) showed a greater diuretic effect added to furosemide compared with furosemide alone, but tolvaptan did not have a positive impact on other clinical outcomes, such as length of hospital stay or postdischarge outcomes.^39^

The results of clinical trials conducted to date that have analyzed the administration of HSS are consistent with real‐life data published in recent years that have shown a greater diuretic effect, fewer electrolyte disturbances, and a good safety profile.^8^, ^40^, ^41^ It has previously been suggested that the number of unknowns is important for the implementation of HSS in clinical practice.^42^ What should be the optimal dose of HSS? How should sodium and fluid intake be restricted? What criteria should be used to select patients who would benefit from this intervention? These questions should be addressed in new clinical trials that specifically evaluate these scenarios. Based on the findings of our review, we propose that the clinical trial should meet the following characteristics to make a better recommendation considering the greatest benefit of the intervention: (i) the study population should be hospitalized patients with ADHF, NYHA functional class III–IV, NT‐proBNP levels ≥300 pg/mL, refractory to initial intravenous therapy with loop diuretics, serum creatinine levels >2 mg/dL and systolic blood pressure >90 mmHg; (ii) HSS plus furosemide should be initiated in the emergency when the patient is at highest clinical congestion; (iii) the composite primary outcome would be the combination of rehospitalization for heart failure or all‐cause mortality during the 6‐month follow‐up and the secondary outcomes would be the change in the clinical congestion score, urinary output, and serum creatinine measured at admission and at 72 h; and (iv) according to the results of the TSA analysis, the sample size should be at least 1000 patients.

In view of the available evidence, the following question should be raised: what could HSS offer compared to other conventional therapies for congestion in ADHF? First, fewer adverse effects were seen with HSS added to standard high‐dose loop diuretic therapy, including fewer electrolyte abnormalities and lesser renal impairment. Second, there are data that point to higher efficacy of HSS treatment, including a reduced length of hospital stay and faster resolution of congestion. Furthermore, the cost of HSS therapy is possibly a more cost‐effective option than other ADHF therapies (including new diuretics or renal replacement therapy), and the shorter length of hospital stay would also contribute to a reduction in healthcare costs. However, the administration of HSS in ADHF patients should not be undertaken indiscriminately, and it is necessary to properly select appropriate candidate patients that may potentially benefit from this therapy, for instance, those with serum sodium levels in the low range, or high risk of developing diuretic resistance. Based on the results of the intermediate outcomes, the use of HSS plus furosemide could be useful in ADHF patients with functional class NYHA III–IV and without response to initial diuretic therapy. The HSS concentration should be in accordance with the serum Na+ levels. Regarding the dose of furosemide, although several trials used high doses, the subgroup analysis did not show significant differences between doses higher and lower than 200 mg per day. Therefore, a lower dose could be used.

There are two previously published systematic reviews that evaluated the effect of HSS plus furosemide in ADHF patients (Supporting Information: Table S8).^43^, ^44^ Gandhi et al.^44^ performed a meta‐analysis of 10 RCTs published up to 2013. However, they erroneously pooled two related trials in which one was a follow‐up to the other.^20^, ^38^ In addition, the risk of bias was assessed using the Newcastle‐Ottawa Scale even though the tool was designed to be applied only in observational studies. In contrast, we conducted the risk of bias assessment using the most up‐to‐date version of the Cochrane tool for assessing RCTs. Covic et al.^43^ performed a meta‐analysis of 12 studies published up to 2020, combining data from observational studies and RCTs. The combination of these two study designs is not recommended because they could potentially lead to misleading results. Instead, our review focused only on fully published RCTs. Furthermore, we performed a trial sequential analysis to assess whether our results were sufficiently powered to produce firm conclusions about the efficacy of HSS plus furosemide. In fact, our review is the only one that evaluated the quality of evidence for all outcomes using the GRADE approach. Overall, the relevance of our meta‐analysis lies in that we used state‐of‐the‐art methods to yield reliable conclusions overcoming the methodological problems of previous reviews.

This study has some limitations that should be acknowledged. First, meta‐analysis of all‐cause mortality, cardiovascular mortality, readmission for heart failure was not conducted, and only a narrative synthesis was performed. Although none of included RCTs were adequately powered to evaluate these clinical outcomes, they showed a signal reduction in mortality and readmissions for heart failure. However, proper evaluation of these “hard” outcomes in future studies is necessary before providing a recommendation on their use in clinical practice since this is a critical aspect for other pharmacological agents (e.g., seleraxin, ularitide, nesiritide, or milrinone) previously studied in patients with ADHF.^45^ Second, many studies included in our review come from a single healthcare system from on country (Italy). However, the meta‐analysis of non‐Italian trials was consistent with the main analysis. Third, although some studies included a small number of patients, the TSA‐adjusted results showed firm evidence of benefit. Fourth, the observed benefit on hospitalization time may have been overestimated considering that most of the studies conducted in Italy presented longer hospitalization time at baseline. In addition, detailed information on the site (emergency department or hospital ward) where the patients were treated is not available in most studies in Italy. However, the direction of the effect is consistent among the included trials and the subgroup analysis showed no significant difference on hospitalization time when the Italian studies were excluded. Fifth, between‐study heterogeneity was important for most outcomes. This may be explained by the type of population evaluated (different severity of clinical congestion), different timing of outcome assessment, and heterogeneous HSS protocol. This component was considered into the GRADE evaluation. Finally, most studies included patients with heart failure and reduced LVEF (<40%). It would be of interest to analyze in the future whether our findings are also applied to patients with heart failure and preserved ejection fraction.

## 5 CONCLUSIONS

Our meta‐analysis suggests that HSS plus furosemide compared to furosemide alone improves surrogate outcomes in ADHF patients based on low to moderate quality of evidence. However, further adequately powered RCTs are still needed to confirm our findings and to assess the effect on heart failure readmissions and mortality.

## AUTHOR CONTRIBUTIONS

Carlos Diaz‐Arocutipa, Jack Denegri‐Galvan, and Adrian V. Hernandez involved in concept/design. Carlos Diaz‐Arocutipa, Jack Denegri‐Galvan, and Adrian V. Hernandez involved in data acquisition. Carlos Diaz‐Arocutipa, Jack Denegri‐Galvan, and Adrian V. Hernandez involved in data analysis/interpretation. Carlos Diaz‐Arocutipa drafted the article. Jack Denegri‐Galvan, Lourdes Vicent, Marcos Pariona, Mamas A. Mamas, and Adrian V. Hernandez critically revised the article. Carlos Diaz‐Arocutipa, Jack Denegri‐Galvan, Lourdes Vicent, Marcos Pariona, Mamas A. Mamas, and Adrian V. Hernandez approved the article.

## CONFLICT OF INTEREST STATEMENT

The authors declare no conflict of interest.

## Supporting information

Supporting information.

## Data Availability

The data that support the findings of this study are available from the corresponding author upon reasonable request.
