# Supplementary material for: The added value of hypertonic saline solution to furosemide monotherapy in patients with acute decompensated heart failure: A meta‐analysis and trial sequential analysis
Source: Clin Cardiol. 2023 Jun 20;46(8):853–65. doi: 10.1002/clc.24033 (PMC10436795; doi:10.1002/clc.24033)
Supplement: Supplementary file 1 — Supporting information. [file CLC-46-853-s001.docx]

**SUPPLEMENTARY MATERIAL**

**Table S1. Search strategy**

| *PubMed:*  (hypersaline[tiab] OR “hypertonic saline”[tiab] OR “Saline Solution, Hypertonic”[mesh]) AND (“heart failure”[mesh] OR “heart failure”[tiab] OR “heart insufficiency”[tiab] OR “cardiac failure”[tiab] OR “cardiac insufficiency”[tiab]) |
| --- |
| *Embase:*  (hypersaline:ti,ab OR “hypertonic saline”:ti,ab) AND (“heart failure” OR “heart insufficiency” OR “cardiac failure” OR “cardiac insufficiency”) |
| *Scopus:*  TITLE-ABS-KEY ((hypersaline OR “hypertonic saline”) AND (“heart failure” OR “heart insufficiency” OR “cardiac failure” OR “cardiac insufficiency”)) |
| *Web of Science:*  TS=((hypersaline OR “hypertonic saline”) AND (“heart failure” OR “heart insufficiency” OR “cardiac failure” OR “cardiac insufficiency”)) |

**Table S2. Timing of outcome assessment for all trials**

| Study | All-cause mortality | CV mortality | HF readmission | Weight | Urine output | Serum creatinine | Serum Na^+^ | Urine Na^+^ | BNP |
| --- | --- | --- | --- | --- | --- | --- | --- | --- | --- |
| Licata, 2003 | Mean 31 months | Mean 31 months | Mean 31 months | At discharge | At discharge | At discharge | At discharge | At discharge | - |
| Paterna, 2005 | 30 days | 30 days | 30 days | At discharge | At discharge | At discharge | At 6 day | At discharge | At 30 day |
| Parrinello, 2011 | - | - | - | - | At 6 day | At 6 day | At 6 day | At 6 day | - |
| Paterna, 2011 | Mean 57 months | Mean 57 months | Mean 57 months | - | At discharge | At discharge | At discharge | At discharge | At discharge |
| Parrinello, 2012 | - | - | - | - | At discharge | At discharge | At discharge | - | At discharge |
| Issa, 2013 | Discharge | - | - | - | At 24 hours | At 24 hours | At 24 hours | - | At 24 hours |
| Okuhara, 2014 | - | - | - | - | - | At 24 hours | At 24 hours | At 24 hours | - |
| Yayla, 2015 | - | - | - | At 48 hours | - | At 48 hours | At 48 hours | - | - |
| Wan, 2017 | 4 years | 4 years | - | - | At discharge | - | At discharge | - | At discharge |
| Tuttolomondo, 2021 | - | - | - | At 6 day | At 6 day | - | - | - | - |

BNP, B-type natriuretic peptide; CV, cardiovascular; HF, heart failure.

**Table S3. Subgroup analysis according to the type of population**

| Outcome | Number of trials | Sample size | Effect measure (95% CI) | I^2^ | p-value for interaction |
| --- | --- | --- | --- | --- | --- |
| Length of hospital stay |  |  |  |  |  |
| Refractory | 5 | 2525 | MD: -3.45 (-4.49 to -2.40) | 94% | 0.39 |
| Unselected | 2 | 276 | MD: -3.97 (-4.53 to -3.40) | 0% |  |
| Weight |  |  |  |  |  |
| Refractory | 2 | 201 | MD: 2.09 (0.72-3.46) | 53% | 0.50 |
| Unselected | 2 | 164 | MD: 2.69 (1.63-3.75) | 4% |  |
| Urine output |  |  |  |  |  |
| Refractory | 5 | 2525 | MD: 494 (445.97-542.68) | 6% | 0.80 |
| Unselected | 2 | 384 | MD: 542.82 (173.76-911.88) | 95% |  |
| Serum creatinine |  |  |  |  |  |
| Refractory | 4 | 2261 | MD: -0.52 (-0.59 to --0.45) | 95% | <0.01 |
| Unselected | 4 | 352 | MD: -0.04 (-0.38 to 0.29) | 85% |  |
| Serum Na^+^ |  |  |  |  |  |
| Refractory | 5 | 2525 | MD: 8.99 (6.30 to 11.68) | 98% | 0.04 |
| Unselected | 4 | 352 | MD: 3.50 (-0.97 to 7.97) | 94% |  |
| Urine Na^+^ |  |  |  |  |  |
| Refractory | 4 | 2261 | MD: 54.85 (46.31-63.38) | 94% | - |
| Unselected | - | - | - | - |  |
| BNP |  |  |  |  |  |
| Refractory | 3 | 2285 | MD: -127.69 (-224.28 to -31.10) | 99% | 0.48 |
| Unselected | 2 | 280 | MD: 203.10 (-705.16 to 1111.36) | 71% |  |

MD, mean difference; RR, risk ratio; CI, confidence interval; BNP, B-type natriuretic peptide.

**Table S4. Subgroup analysis according to the duration of HSS plus furosemide**

| Outcome | Number of trials | Sample size | Effect measure (95% CI) | I^2^ | p-value for interaction |
| --- | --- | --- | --- | --- | --- |
| Length of hospital stay |  |  |  |  |  |
| ≥6 days | 2 | 240 | MD: -4.39 (-6.90 to -1.87) | 91% | 0.43 |
| <6 days | 5 | 2561 | MD: -3.29 (-4.30 to -2.27) | 95% |  |
| Weight |  |  |  |  |  |
| ≥6 days | 2 | 243 | MD: 2.22 (0.69-3.75) | 68% | 0.77 |
| <6 days | 2 | 122 | MD: 2.50 (1.33-3.68) | 0% |  |
| Urine output |  |  |  |  |  |
| ≥6 days | 2 | 376 | MD: 475.67 (295.53-655.81) | 72% | 0.44 |
| <6 days | 4 | 2533 | MD: 564.05 (431.28-696.82) | 82% |  |
| Serum creatinine |  |  |  |  |  |
| ≥6 days | 2 | 240 | MD: -0.62 (-0.76 to -0.47) | 87% | <0.01 |
| <6 days | 6 | 2373 | MD: -0.27 (-0.40 to -0.15) | 90% |  |
| Serum Na^+^ |  |  |  |  |  |
| ≥6 days | 2 | 240 | MD: 10.58 (7.54-13.61) | 86% | <0.01 |
| <6 days | 7 | 2637 | MD: 6.80 (4.92-8.69) | 97% |  |
| Urine Na^+^ |  |  |  |  |  |
| ≥6 days | 2 | 240 | MD: 57.22 (35.72-78.73) | 92% | 0.92 |
| <6 days | 2 | 2021 | MD: 55.57 (30.10-81.03) | 97% |  |
| BNP |  |  |  |  |  |
| ≥6 days | - | - | - | - | - |
| <6 days | 5 | 2565 | MD: -124.26 (-207.97 to -40.54) | 97% |  |

MD, mean difference; CI, confidence interval; BNP, B-type natriuretic peptide.

**Table S5. Subgroup analysis according to the daily dose of furosemide**

| Outcome | Number of trials | Sample size | Effect measure (95% CI) | I^2^ | p-value for interaction |
| --- | --- | --- | --- | --- | --- |
| Length of hospital stay |  |  |  |  |  |
| >200 mg/day | 5 | 2509 | MD: -3.68 (-5.01 to -2.36) | 96% | 0.41 |
| ≤200 mg/day | 2 | 292 | MD: -3.07 (-3.64 to -2.51) | 4% |  |
| Weight |  |  |  |  |  |
| >200 mg/day | 3 | 337 | MD: -2.42 (-3.38 to -1.46) | 43% | 0.53 |
| ≤200 mg/day | 1 | 28 | MD: -1.60 (-3.96 to 0.76) | - |  |
| Urine output |  |  |  |  |  |
| >200 mg/day | 6 | 2645 | MD: 536.92 (420.56 to 653.28) | 80% | 0.56 |
| ≤200 mg/day | 1 | 264 | MD: 486 (362.47 to 609.53) | - |  |
| Serum creatinine |  |  |  |  |  |
| >200 mg/day | 5 | 2509 | MD: -0.49 (-0.56 to -0.42) | 94% | <0.01 |
| ≤200 mg/day | 3 | 104 | MD: 0.14 (-0.04 to 0.33) | 0% |  |
| Serum Na^+^ |  |  |  |  |  |
| >200 mg/day | 5 | 2509 | MD: 9.82 (8.21 to 11.43) | 94% | <0.01 |
| ≤200 mg/day | 4 | 368 | MD: 2.62 (0.98 to 4.26) | 56% |  |
| Urine Na^+^ |  |  |  |  |  |
| >200 mg/day | 4 | 2261 | MD: 54.85 (46.31 to 63.38) | 94% | - |
| ≤200 mg/day | - | - | - | - |  |
| BNP |  |  |  |  |  |
| >200 mg/day | 3 | 2269 | MD: -130.01 (-255.01 to -5.01) | 93% | 0.48 |
| ≤200 mg/day | 2 | 296 | MD: 201.72 (-708.36 to 1111.81) | 71% |  |

MD, mean difference; CI, confidence interval; BNP, B-type natriuretic peptide.

**Table S6. Subgroup analysis according to the country**

| Outcome | Number of trials | Sample size | Effect measure (95% CI) | I^2^ | p-value for interaction |
| --- | --- | --- | --- | --- | --- |
| Length of hospital stay |  |  |  |  |  |
| Italy | 5 | 2509 | MD: -3.68 (-5.01 to -2.36) | 96% | 0.41 |
| Non-Italian countries | 2 | 292 | MD: -3.07 (-3.64 to -2.51) | 4% |  |
| Weight |  |  |  |  |  |
| Italy | 3 | 337 | MD: -2.42 (-3.38 to -1.46) | 43% | 0.53 |
| Non-Italian countries | 1 | 28 | MD: -1.60 (-3.96 to 0.76) | - |  |
| Urine output |  |  |  |  |  |
| Italy | 6 | 2645 | MD: 536.92 (420.56 to 653.28) | 80% | 0.56 |
| Non-Italian countries | 1 | 264 | MD: 486 (362.47 to 609.53) | - |  |
| Serum creatinine |  |  |  |  |  |
| Italy | 5 | 2509 | MD: -0.49 (-0.56 to -0.42) | 94% | <0.01 |
| Non-Italian countries | 3 | 104 | MD: 0.14 (-0.04 to 0.33) | 0% |  |
| Serum Na^+^ |  |  |  |  |  |
| Italy | 5 | 2509 | MD: 9.82 (8.21 to 11.43) | 94% | <0.01 |
| Non-Italian countries | 4 | 368 | MD: 2.62 (0.98 to 4.26) | 56% |  |
| Urine Na^+^ |  |  |  |  |  |
| Italy | 4 | 2261 | MD: 54.85 (46.31 to 63.38) | 94% | - |
| Non-Italian countries | - | - | - | - |  |
| BNP |  |  |  |  |  |
| Italy | 3 | 2269 | MD: -130.01 (-255.01 to -5.01) | 93% | 0.48 |
| Non-Italian countries | 2 | 296 | MD: 201.72 (-708.36 to 1111.81) | 71% |  |

MD, mean difference; CI, confidence interval; BNP, B-type natriuretic peptide.

**Table S7. Sensitivity analysis including only trials with low risk of bias**

| Outcome | Number of trials | Sample size | MD (95% CI) | I^2^ |
| --- | --- | --- | --- | --- |
| Length of hospital stay | 4 | 503 | -4.38 (-5.21 to -3.56) | 57% |
| Serum creatinine | 5 | 535 | -0.30 (-0.52 to -0.08) | 94% |
| Serum Na^+^ | 5 | 535 | 6.94 (3.97-9.91) | 94% |
| Urine Na^+^ | 2 | 227 | 57.55 (36.01-79.09) | 95% |
| Urine output | 3 | 475 | 681.23 (595.09-767.38) | 0% |
| BNP | 3 | 374 | -170.28 (-306.29 to -34.27) | 69% |
| Weight | 2 | 122 | -2.50 (-3.68 to -1.33) | 0% |

MD, mean difference; CI, confidence interval; BNP, B-type natriuretic peptide.

**Table S8. Comparison of published systematic reviews on the use of HSS plus furosemide in patients with acute decompensated heart failure**

|  | Our study | Covic et al. | Gandhi et al. |
| --- | --- | --- | --- |
| Year of publication | 2022 | 2021 | 2014 |
| Aim | To assess the effects of HSS plus furosemide versus furosemide alone in patients with acute decompensated HF | To evaluate the efficiency of HSS plus furosemide therapy in patients with decompensated HF | To assess the efﬁcacy of HSS in combination with furosemide for the treatment of acute advanced congestive HF |
| Search databases | Pubmed, Embase, Scopus, Web of Science | Embase, CENTRAL, PubMed, and Web of Science | PubMed, Embase, and Cochrane |
| Search cut-off date | June 2022 | May 2020 | November 2013 |
| Population | Adult patients with acute decompensated HF | Patients with acute decompensated HF | Patients with acute advanced congestive HF treated with HSS and adjuvant furosemide |
| Included studies | 10 RCTs | 10 RCTs and 4 cohorts | 10 RCTs |
| Total number of patients | 3013 patients | 3398 patients | 2845 patients |
| Risk of bias assessment | Cochrane Risk of Bias 2.0 tool | Cochrane Risk of Bias 1.0 tool and Newcastle–Ottawa Scale | Newcastle–Ottawa Scale |
| GRADE evaluation | Yes | No | No |
| Outcomes | Length of hospital stay, urine output, weight, serum Na^+^, urine Na^+^, serum creatinine, and BNP | Mortality, readmissions, length of hospital stay, serum creatinine, urine output, body weight, and BNP | All-cause mortality, HF hospital readmission, length of hospitalizacion, weight loss, serum creatinine |
| Conclusion | HSS plus furosemide compared to furosemide alone improves outcomes in patients with acute decompensated HF | The intravenous administration of HSS with furosemide in patients with acute decompensated HF may result in shorter mean hospital stays, lower mortality rates, fewer readmissions, and significant improvements in serum creatinine levels, 24-h urine output, and weight loss compared with intravenous furosemide therapy alone | In patients with advanced congestive HF concomitant HSS administration improved weight loss, preserved renal function, and decreased length of hospitalization, mortality and heart failure rehospitalization. |

HF, heart failure; RCT, randomized controlled trial; GRADE, Grading of Recommendations, Assessment, Development and Evaluation; HSS, hypertonic saline solution; BNP, type-B natriuretic peptide.

**Figure S1. Risk of bias assessment**

**
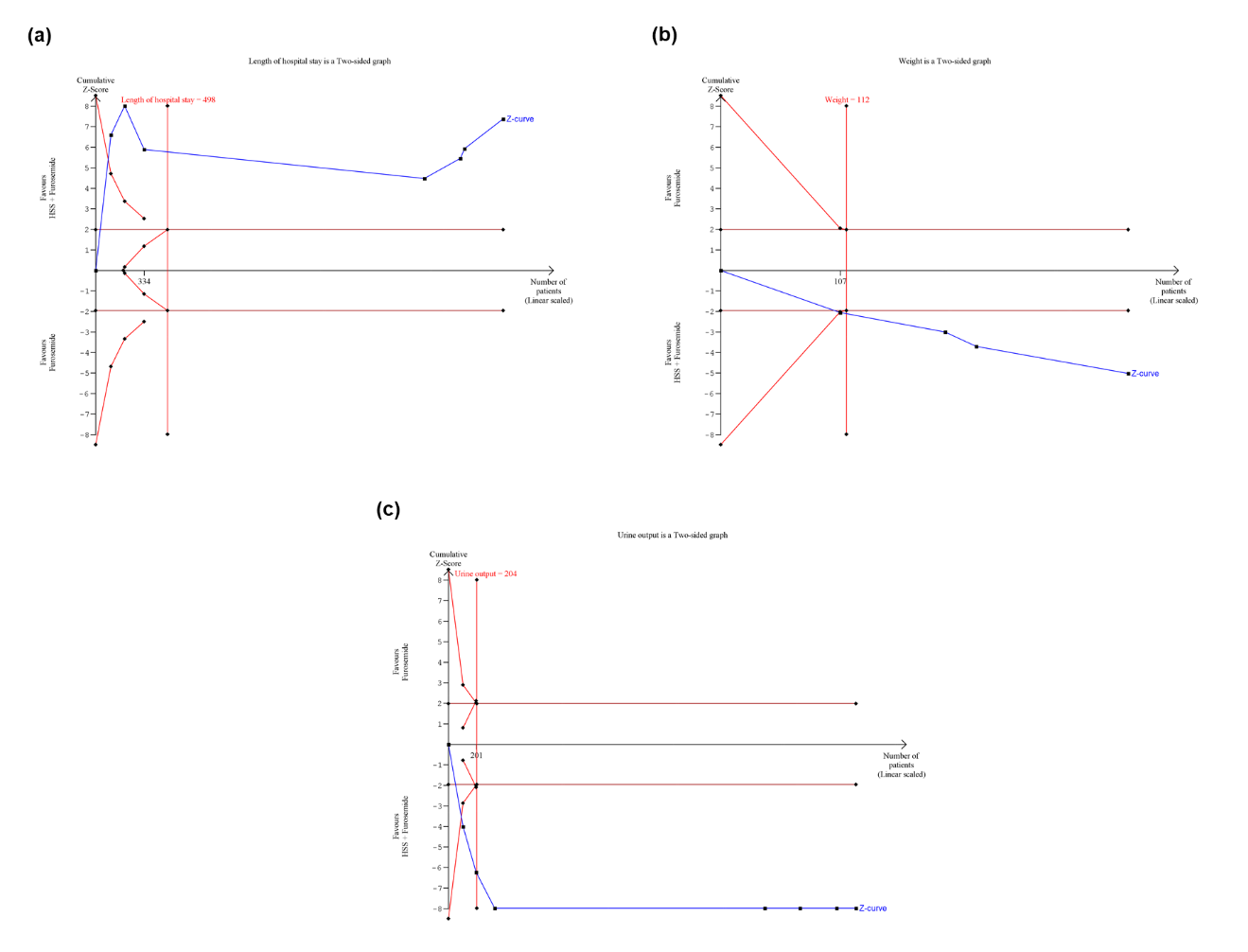
**

**Figure S2. Trial sequential analysis of effects of HSS plus furosemide versus furosemide on (a) length of hospital stay (TSA-adjusted CI: -4.83 to -2.37), (b) weight (TSA-adjusted CI: -3.25 to -1.39), and (c) urine output (TSA-adjusted CI: 430.25 to 626.88). Abbreviations: HSS, hypertonic saline solution; TSA, trial sequential analysis; CI, confidence interval.**


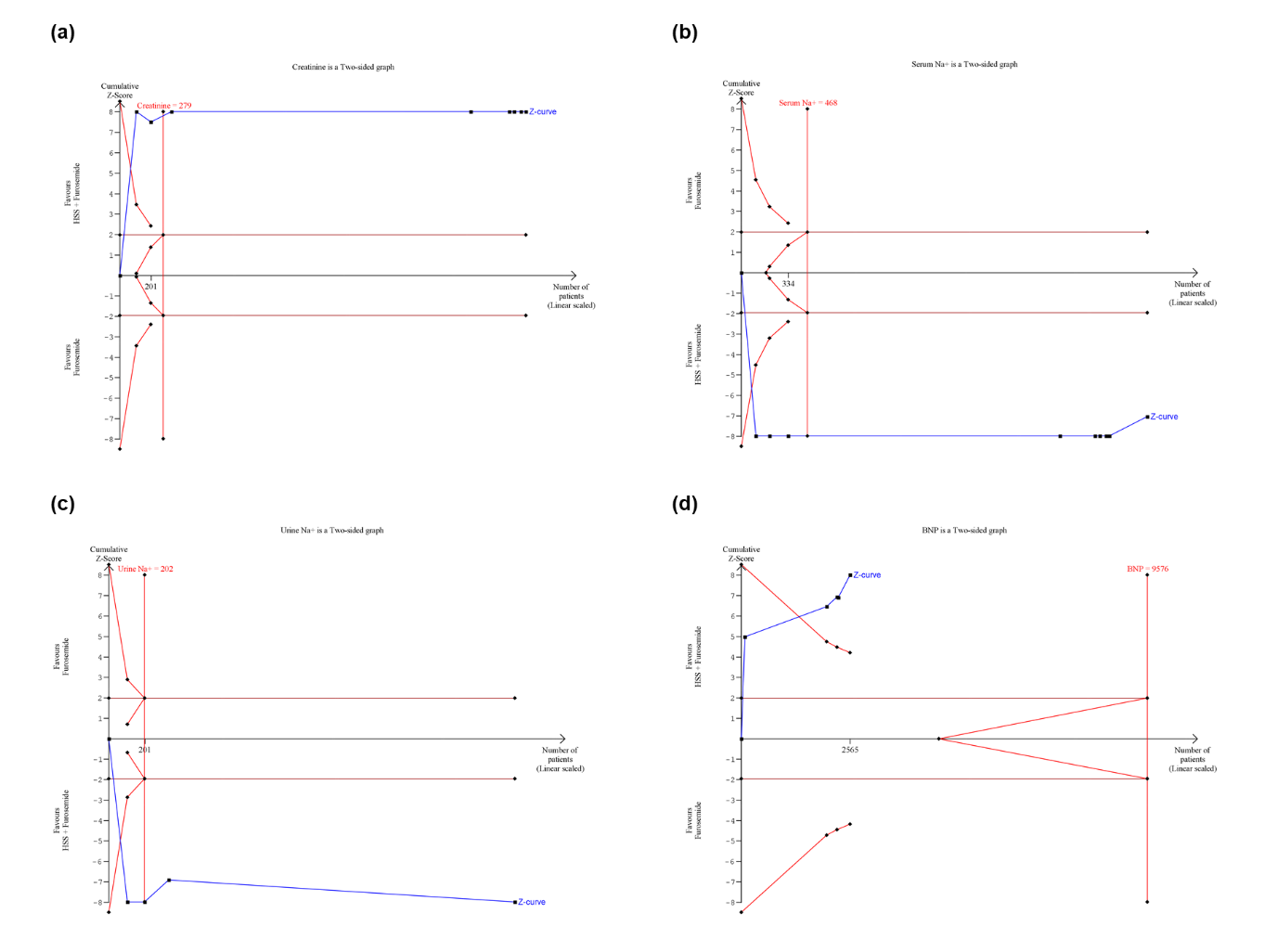


**Figure S3. Trial sequential analysis of the effects of HSS plus furosemide versus furosemide on (a) serum creatinine (TSA-adjusted CI: -0.51 to -0.31), (b) serum Na^+^ (TSA-adjusted CI: 4.48 to 9.13), (c) urine Na^+^ (TSA-adjusted CI: 46.23 to 63.46), and (d) BNP (TSA-adjusted CI: -79.93 to -44.20). Abbreviations: HSS, hypertonic saline solution; TSA, trial sequential analysis; CI, confidence interval; BNP, B-type natriuretic peptide.**


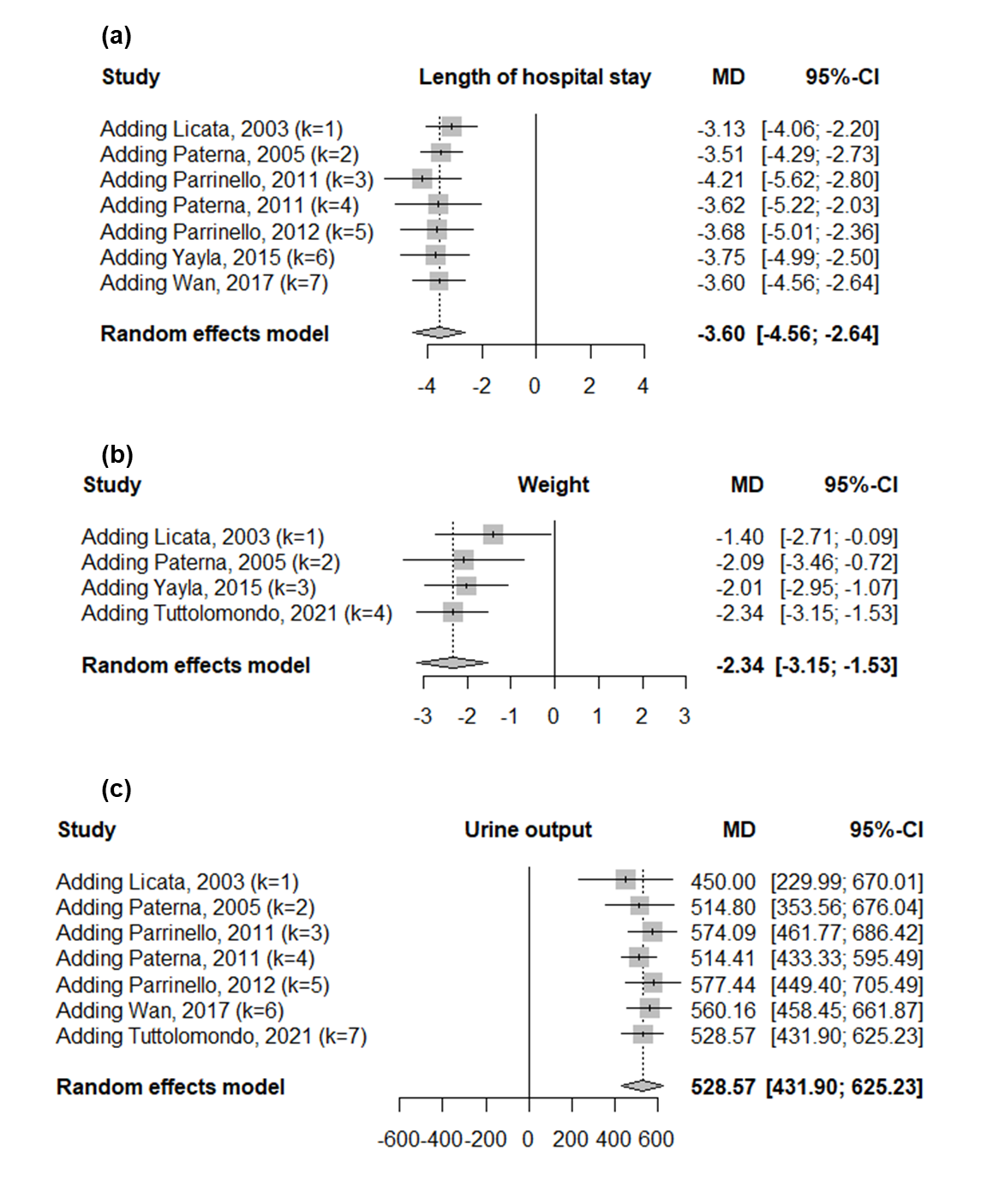


**Figure S4. Cumulative meta-analysis comparing the effects of HSS plus furosemide versus furosemide on (a) length of hospital stay, (b) weight loss, and (c) urine output. Abbreviations: HSS, hypertonic saline solution; MD, mean difference; CI, confidence interval.**

**
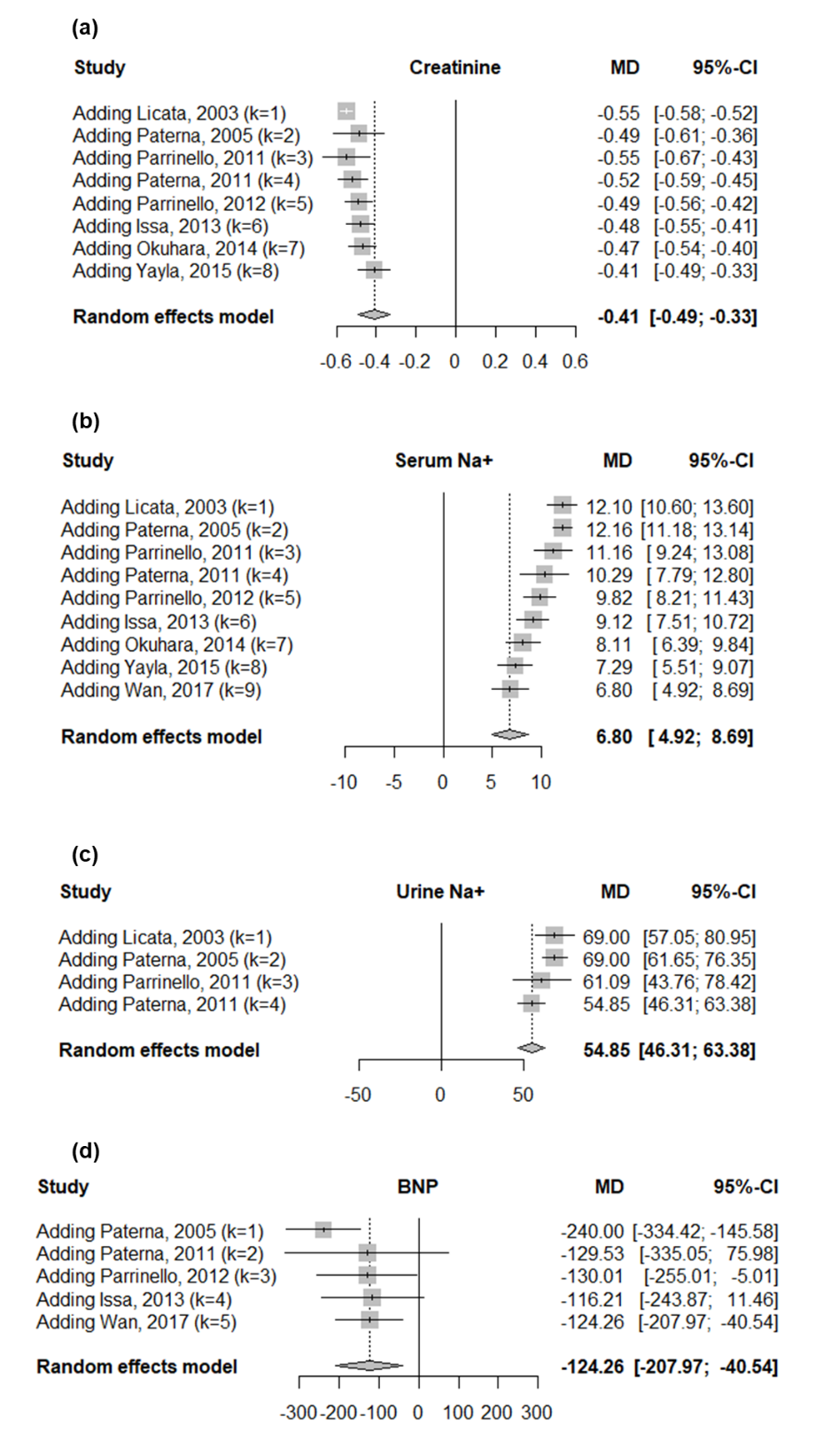
**

**Figure S5. Cumulative meta-analysis comparing the effects of HSS plus furosemide versus furosemide on (a) serum creatinine, (b) serum Na^+^, (c) urine Na^+^, (d) BNP, and (e) systolic blood pressure. Abbreviations: HSS, hypertonic saline solution; MD, mean difference; CI, confidence interval; BNP, B-type natriuretic peptide.**
